# Hay-bait traps are a useful tool for sampling of soil dwelling millipedes and centipedes

**DOI:** 10.3897/zookeys.510.9020

**Published:** 2015-06-30

**Authors:** Ivan H. Tuf, Vojtěch Chmelík, Igor Dobroruka, Lucie Hábová, Petra Hudcová, Jan Šipoš, Slavomír Stašiov

**Affiliations:** 1 Department of Ecology and Environmental Sciences, Faculty of Science, Palacký University, Šlechtitelů 11, 77900 Olomouc, Czech Republic; 2 Department of Biology and Ecology, Faculty of Science, University of Ostrava, Chittusiho 10, 71000 Slezská Ostrava, Czech Republic; 3 Global Change Research Centre, Academy of Sciences of the Czech Republic, Bělidla 986/4a, 603 00 Brno, Czech Republic; 4 Department of Biology and General Ecology, Faculty of Ecology and Environmental Sciences, Technical University in Zvolen, T. G. Masaryka 24, 96053 Zvolen, Slovakia

**Keywords:** Diplopoda, Chilopoda, soil sampling, agroecosystem, soil fauna

## Abstract

Some species of centipedes and millipedes inhabit upper soil layers exclusively and are not recorded by pitfall trapping. Because of their sensitivity to soil conditions, they can be sampled quantitatively for evaluation of soil conditions. Soil samples are heavy to transport and their processing is time consuming, and such sampling leads to disturbance of the soil surface which land-owners do not like. We evaluated the use of hay-bait traps to sample soil dwelling millipedes and centipedes. The effectiveness of this method was found to be similar to the effectiveness of soil sampling. Hay-bait traps installed for 8–10 weeks can substitute for direct soil sampling in ecological and inventory studies.

## Introduction

Soil macrofauna is commonly used for monitoring or evaluation of sites. Besides ground beetles (e.g. Hůrka et al. 1996, Kotze et al. 2011), spiders (e.g. Buchar and Růžička 2002, Maelfait et al. 2004) or woodlice (e.g. Souty-Grosset et al. 2005, Tuf and Tufová 2008), centipedes and millipedes are sampled frequently too (Tuf and Tufová 2008, Dunger and Voigtländer 2009). Nevertheless, not all species of centipedes and millipedes are ground-dwelling with many species inhabiting the upper soil layer exclusively (Lee 2006, Barber and Keay 1988). Soil dwelling animals can be sampled using litter/soil sieving, soil sampling or hand-collecting. Sieved material and soil samples have to be hand-sorted or processed using heat extractors, e.g. Tullgren funnel or Kempson apparatus (Tuf and Tvardík 2005). Handling of soil samples can be difficult due to the higher weight of samples (one sample of size 25 × 25 × 10 cm weighs around 6 kg). Litter/soil sieving can reduce the weight of samples, nevertheless as with hand-collecting, it is time consuming and attention-intensive. Moreover, soil sampling can cause damage to the site; pot-holes created by a soil corer can endanger people passing the site and can increase water erosion on slopes. These pot-holes are definitely not popular among land-owners of the sampled sites. For these reasons (severity of sampling, damage of ground), we have attempted to evaluate the effectiveness of sampling centipedes and millipedes using hay-bait traps. The aims of this research were 1) comparison of the efficiency of hay-bait trapping, soil sampling and pitfall trapping and 2) to find the optimal length of exposure of hay-bait traps for maximum efficiency.

## Material and methods

### Field study

The research was done at three sites in the Czech Republic from May to July 2013. The first site was an alfalfa field (49°34.41'N, 17°17.17'E) on the border of the town of Olomouc. This large field of ca 250 square metres is surrounded by other fields (with cereals) and a railway embankment. In the previous year it had also grown alfalfa. The field is under conventional management including use of herbicides and ploughing.

The second site was an old meadow (50°26.85'N, 15°0.00'E) being mown once to twice per year for the last 30 years. This meadow of ca 500 square metres is surrounded by fields and gardens with mixed wood across the road and is ca 6 km north-east of the town of Mladá Boleslav. The third site studied was a mixed forest (49°15.66'N, 17°17.72'E) 6 km south-west of the town of Kroměříž. The forest is classified as *Fageto*-*Quercetum illimerosum trophicum*; dominant trees are oaks, hornbeams and some pines, with *Rubus fruticosus*, *Galium odoratum* and *Galium aparine* as dominants of undergrowth. The soil surface of this forest is covered by a rather thick layer of oak leaf litter.

In the Czech Republic generally, the weather conditions during the study period were characterised by average or slightly increased temperatures and higher than average precipitation in May-June, and a very hot July in contrast to long-term average values. The previous winter season was rather warmer and with higher precipitation (ref. historical territorial data at www.chmi.cz).

Soil macrofauna, including millipedes and centipedes, was sampled using three methods at each site. Pitfall traps (10 traps consisting of glass jars with inserted plastic pots of diameter 7.5 cm filled with 2 dl of 4% formaldehyde in water with some detergent, metal covers) were arranged in 2 lines of 5 traps with a span of 10 m, and inspected at 2-week intervals. Five soil samples (25 × 25 × 10 cm including litter layer) were obtained using a spatula, three times per study (i.e. 15 soil samples per site) and transported to the laboratory in plastic bags. Hay-bait traps were made from a wire gauze (2 cm mesh) shaped as a simple pocket of size 25 × 25 cm. Each pocket was marked by a code written on the band. These pockets were filled with hay (commercial hay mixture for feeding rodent pets) and submerged into water for 2 hours before installation. Altogether, 60 hay-bait traps were placed horizontally at each site in a following scheme: 5 lines of 12 traps (2-5 cm under soil surface) over a length of 2 m with 10 m between lines. All traps were installed at the same time and 5 traps were taken away each week during the course of the study lasting for 12 weeks. Hay-traps were transported into the laboratory inside separate plastic bags.

### Sample processing

Soil samples and hay-traps were heat-extracted immediately in the laboratory using simple Kempson devices (Tuf and Tvardík 2005). Hay-traps were extracted for a week, soil samples for 2 weeks, both under electric 60W-bulbs. Extracted animals from both soil samples and hay-traps were sorted to higher taxonomic groups and millipedes and centipedes were identified to the species level.

### Data analyses

We tested the effects of trapping time and methods on species richness by repeated-measures on traps with nested design. The traps were nested in each of the three study sites (field, meadow, forest). Explanatory variables in the model were trapping time and trapping methods. The response variable was defined as a number of species per trap for particular time and place. Habitat type was used as random variable. We used a mixed model to estimate the correct error term and degrees of freedom. To test this effect, a generalized linear mixed model (glmmPQL, part of R package MASS) was used with negative binomial error distribution and log link function (Bates et al. 2014).

To test if one level of a particular factor (trapping method and study site) is more variable than other levels of the same factor, a permutation test was used (permutest.betadisper, part of R package vegan). This permutation based method tests pairwise comparisons of group mean dispersions. It is based on the t-statistic computed on pairwise group dispersions. A distance matrix was computed based on “Bray-Curtis” index of dissimilarity (vegdist, part of R package vegan). Then the function “betadisper” (part of vegan package in R) was used to calculate variance for each group of samples. Variance was computed as average distance of group members to the group centroid.

Rarefaction curves were constructed to show how the species richness varies for the same sample size between the three trapping methods. Function “rarefy” (part of vegan package in R software) computed the expected species richness and standard deviation in random subsamples of a particular sample size from the community. Data were analysed using R software (R Development Core Team 2011).

## Results

Altogether, we obtained 541 millipedes from 17 species and 435 centipedes from 13 species (Table 1). Based on the number of recorded animals, the richest site was the forest (553 myriapods) and poorest site was the field (100 myriapods). Number of species showed the same pattern: 21 myriapod species in the forest and 6 in the field. Soil sampling was the least efficient for sampling species (9 millipede and 7 centipede species) as well as individuals (36 and 100 individuals respectively), whereas pitfall traps and hay-bait traps were similar in their efficiency: 14–15 millipede and 9–10 centipede species; for number of individuals, see Table 1.

**Table 1.** List of millipedes obtained using three methods from three biotopes (ind./10 pitfall traps/12 weeks, ind./60 bait traps and ind./0.94m^2^ respectively.

	**Pitfall traps**			**Hay-bait traps**			**Soil samples**			**Total pitfall traps**	**Total hay-bait traps**	**Total soil samples**
	**field**	**meadow**	**forest**	**field**	**meadow**	**forest**	**field**	**meadow**	**forest**			
*Glomeris connexa* C. L. Koch, 1847	-	9	1	-	-	1	-	-	-	10	1	0
*Blaniulus guttulatus* (Fabricius, 1798)	-	2	-	-	31	1	-	2	-	2	32	2
*Brachyiulus bagnalli* (Curtis, 1845)	2	-	-	5	-	-	-	-	-	2	5	0
*Cylindroiulus boleti* (C.L. Koch, 1847)	-	-	3	-	-	-	-	-	-	3	0	0
*Cylindroiulus caeruleocinctus* (Wood, 1864)	1	-	-	-	-	-	-	-	-	1	0	0
*Enantiulus nanus* (Latzel, 1884)	-	-	64	-	-	32	-	-	4	64	32	4
*Julus scandinavius* Latzel, 1884	-	-	-	-	-	1	-	-	-	0	1	0
*Leptoiulus proximus* (Němec, 1896)	-	-	2	-	-	1	-	-	-	2	1	0
*Megaphyllum projectus* Verhoeff, 1894	-	-	2	-	-	2	-	-	-	2	2	0
*Ommatoiulus sabulosus* (Linnaeus, 1758)	-	-	10	-	-	11	-	-	4	10	11	4
*Ophiulus pilosus* (Newport, 1842)	27	-	-	59	-	-	2	-	-	27	59	2
*Unciger foetidus* (C.L. Koch, 1838)	-	9	36	-	30	26	-	3	2	45	56	5
*Brachydesmus superus* Latzel, 1884	-	-	-	-	-	-	-	3	-	0	0	3
*Polydesmus complanatus* (Linnaeus, 1761)	-	3	1	-	2	3	-	-	1	4	5	1
*Polydesmus denticulatus* C.L. Koch, 1847	-	8	-	-	7	-	-	-	-	8	7	0
*Polydesmus inconstans* Latzel, 1884	-	1	-	-	39	-	-	6	-	1	39	6
*Strongylosoma stigmatosum* (Eichwald, 1830)	-	-	47	-	-	26	-	-	9	47	26	9
**Diplopoda**	30	32	166	64	109	104	2	14	20	228	277	36
*Clinopodes flavidus* C.L. Koch, 1847	-	-	2	1	-	9	-	-	-	2	10	0
*Geophilus electricus* (Linnaeus, 1758)	-	-	-	-	-	-	-	9	-	0	0	9
*Geophilus flavus* (DeGeer, 1778)	-	-	10	-	9	20	1	30	-	10	29	31
*Geophilus truncorum* Bergsoe & Meinert, 1866	-	-	-	-	-	-	-	1	-	0	0	1
*Schendyla nemorensis* (C.L. Koch, 1836)	-	-	23	-	11	60	-	26	-	23	71	26
*Strigamia transsilvanica* (Verhoeff, 1928)	-	-	4	-	-	2	-	-	-	4	2	0
*Lithobius aerugineus* L. Koch, 1862	-	-	41	-	-	39	-	-	-	41	39	0
*Lithobius austriacus* (Verhoeff, 1937)	-	-	-	-	-	2	-	-	-	0	2	0
*Lithobius dentatus* C.L. Koch, 1844	-	-	2	-	-	1	-	-	-	2	1	0
*Lithobius erythrocephalus* C.L. Koch, 1847	-	-	1	-	-	-	-	-	-	1	0	0
*Lithobius forficatus* (Linnaeus, 1758)	-	-	-	-	-	1	-	-	1	0	1	1
*Lithobius microps* Meinert, 1868	-	4	-	-	47	-	-	31	-	4	47	31
*Lithobius mutabilis* L. Koch, 1862	-	-	3	-	-	3	-	-	1	3	3	1
*Lithobius* spp.	-	-	14	2	-	24	-	-	-	14	26	0
**Chilopoda**	0	4	100	3	67	161	1	97	2	104	231	100

**Figure 1.**
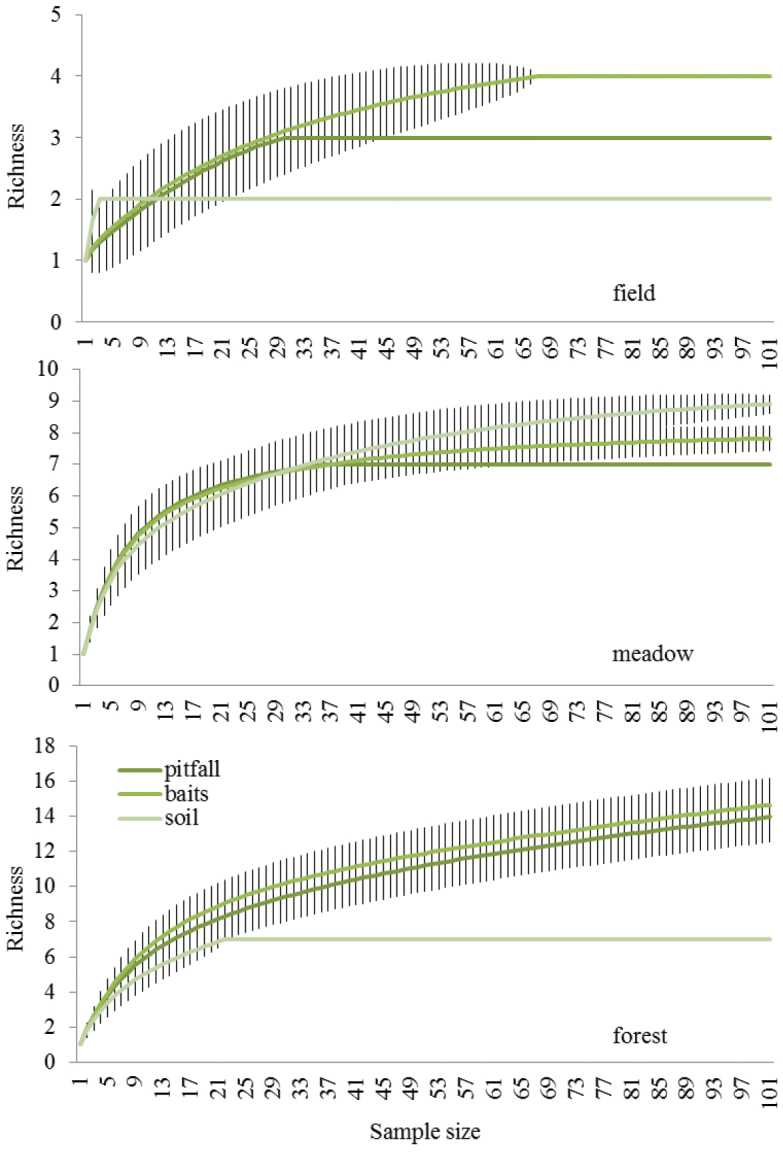
Rarefactions of estimated species richness (i.e. number of species) in increasing size of random samples (i.e. number of individuals), comparison of effectiveness of sampling by individual methods at different sites. Vertical lines represent standard errors.

Methods at individual sites were evaluated according to their efficiency using rarefactions (Fig. 1). Bait traps sampled higher numbers of species in contrast to other methods in the field site, meanwhile increasing sampling effort (number of sampled animals) was connected with a bigger species list in bait traps as well as in pitfall traps in forest.

Differences between species lists at all sites and lists sampled by individual methods were compared by pairwise comparisons and differences confirmed between all pairs of sites (Table 2a). Nevertheless, the same analysis revealed there was no statistically significant difference between the suite of species sampled by hay-bait traps and soil samples (Table 2b).

**Table 2.** Pairwise comparisons of species lists collected (a) at different sites and (b) by different methods. (Observed p-value below diagonal, permuted p-value above diagonal).

a)	field	forest	meadow	b)	hay-bait	pitfall	soil
field	-	0.001	0.048	hay-bait	-	0.003	0.917
forest	0.000	-	0.001	pitfall	0.003	-	0.043
meadow	0.041	0.004	-	soil	0.911	0.052	-

**Figure 2.**
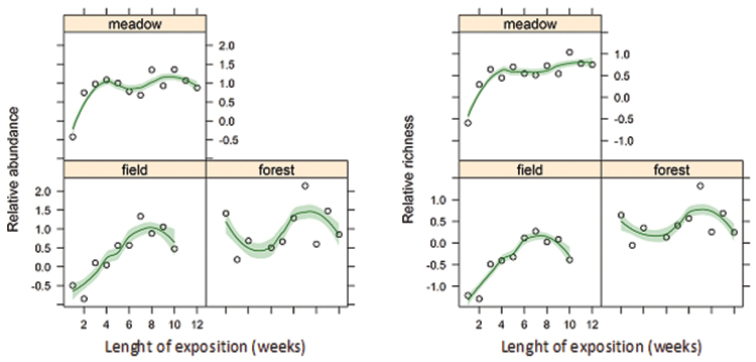
Changes in myriapod communities inside hay-bait traps installed in three biotopes during the 12 week trapping period. Qualitative as well as quantitative parameters are shown for these communities. Open dots are observed parameters, whereas solid lines represent models of succession including standard errors (green shading).

Evaluation of colonization of hay-bait traps (Fig. 2) showed that the highest diversity as well as abundance of collected myriapods in these traps is after 7 weeks following installation in field, or 9-10 weeks following installation in forest or meadow. A longer period of exposure leads to a decrease of both parameters of myriapod communities. Generalized linear mixed models reveal that changes in abundance during exposure was significantly influenced by the second power of time (LRT = 6.43, p = 0.040, AIC = 667.83). The analogous model for diversity confirmed significant changes during time (LRT = 5.81, p = 0.042, AIC = 543.38) which were site dependent too (LRT = 6.74, p = 0.034, AIC = 544.12).

## Discussion

Centipedes and millipedes live on the soil surface and inside soil. We can find them through the whole soil gradient to a depth of one meter (e.g. Ilie 2003) although they are abundant in upper layers predominantly. This is the reason why pitfall traps are not sufficient for sampling the whole community adequately. We evaluated efficiency of hay-bait traps for sampling soil-dwelling millipedes and centipedes with the time consuming soil sampling (connected with destruction of the soil surface and transport of heavy samples to the laboratory).

Bait traps were used for sampling invertebrates, mainly beetles, in caves originally (Barber 1931). Bait traps are much more common for carnivorous or necrophagous species; baits are represented by pieces of flesh, fish or cheese above a fixation solution surface, or direct addition of beer to a solution. Straw, wood or yeast is placed in caves occasionally as the baits for detritivores (Mock pers. comm.). Nevertheless, baits are not working there as traps, as they need to be visited and inspected continuously to collect attracted animals to avoid them to leave baits.

The first documented version of bait traps for millipedes was a shingle trap by Barber (1997) filled with kitchen tissue and potatoes. He used this trap to sample millipedes and isopods at a shingle beach in England. Similar kinds of bait traps, containing sweet potatoes or corn, were used by Brunke et al. (2012) for sampling *Cylindroiulus caeruleocinctus* in Canadian fields. Almost the same traps are used with the name litter bags for studying decomposition of different kinds of litter and/or by different size groups of decomposers (according to diameters of holes in the traps). Litter bags are also used for sampling soil mesofauna or microarthropods (e.g. Wiegert 1974). Prasifka et al. (2007) used litter bags to sample ground dwelling invertebrates; they installed litter bags at the soil surface as well as below the soil surface in a corn field. Above-ground bait traps were attractive for centipedes (millipedes were not recorded in this research). Apart from these publications, we did not found any records of the use of bait traps for sampling millipedes or centipedes.

### Hay-bait traps vs. soil samples

If we are interested in using hay-bait traps as an adequate (or even better) substitute for soil sampling, we have to compare species lists of millipedes and centipedes trapped by these methods. There were only three species recorded exclusively from soil sampling, i.e. missing in hay-bait traps: millipede *Brachydesmus superus* and centipedes *Geophilus electricus* and *Geophilus truncorum*. The minute millipede species lives preferably in clay soils with litter (Lee 2006) usually in huge quantities. This species is a dominant species recorded by pitfall traps in cities (Riedel et al. 2009), so its absence in pitfall traps at the meadow site is probably caused by its low abundance. *Geophilus truncorum* was recorded once only, so it is hard to evaluate effectiveness of sampling of so “rare” a species. Nevertheless, both geophilomorphs (*Geophilus electricus* and *Geophilus truncorum*) are known as predators of earthworms (Sergeeva et al. 1985, Keay 1986); for this reason, hay-bait may be not attractive for them as they follow earthworms into their corridors in soil. So, to collect *Geophilus electricus*, soil sampling or direct hand collecting seems to be necessary. Other geophilomorphs (*Clinopodes flavidus*, *Schendyla nemorensis*, *Schendyla acuminata*, *Geophilus flavus*) are common species, which are frequently found by individual hand-collecting; they live in soil near the surface, under logs, bark and stones (e.g. Barber and Keay 1988). Their presence in shallow hay-bait traps is not surprising as these species were sampled by pitfall trapping too.

One millipede species, *Julus scandinavius* was recorded exclusively in a hay-bait trap, but as one specimen only was found no generalization can be made. Many more species were found in both hay-bait traps and pitfall traps but not in soil samples. Nevertheless, hay-bait traps are not a substitute method to pitfall trapping as there were significant differences between species lists recorded by these methods (see Tab. 2), but it can definitely substitute the soil samples.

### Colonisation of hay-bait traps

Centipedes, and especially millipedes, are attracted into the hay-bait traps. The possible reason can be as a food source and/or sustainable shelter with higher humidity. At least for millipedes, food source seems to be the more probable explanation; wet cloth method (offering higher humidity) did not record any millipedes in African savannah ecosystems (Druce et al. 2004). More probably, millipedes and centipedes are attracted by food availability, as it can be associated with hay decomposition and colonization of the traps. Smaller decomposers colonising baits are welcomed food for carnivorous centipedes (e.g. Perry et al. 1997).

Eight to ten weeks seems to be the optimal exposure time for hay-bait traps in Central European conditions. A similar result was found by Ožanová (2001) using grass traps (a small heap of mowed grass on the surface of meadow), with a much higher number of species after 7 weeks than for a shorter exposure time. Although Prasifka et al. (2007) did not evaluate the effect of length of exposure time of bait traps, it is evident from their results that below-ground traps were more effective after 8 weeks than after 6 weeks. It supports our results that the best length of exposure of bait traps is from 8 to 10 weeks, although we aware of difficulties with this generalization. The best length of exposure is not dependent only on a type of habitat, but also on climate conditions (rainy or dry weather) and time of year when exposed. Traps installed in Central European conditions in late autumn or in winter or during dry hot summer can be colonised in different ways as invertebrates change their activity and position in the soil profile during the year (David 1984, Geoffroy 1985, David et al. 1996, Tuf 2002). The general recommendation for using these traps when installed in spring is to use them for 8–10 weeks. Timing of installation and the length of time exposed in field sites will need to coordinate with agricultural activities such as sowing and harvest times.

## Conclusion

Centipedes and millipedes inhabit the soil surface as well as the soil profile. For a complete knowledge of myriapod fauna, pitfall trapping needs to be combined with a method to collect soil dwelling species, e.g. soil sampling. Hay-bait traps were tested for their ability to replace soil sampling. Our results showed that hay-bait traps are attractive to myriapods and can have a similar sampling effort as soil sampling. The optimal length of exposure of hay-bait traps in soil seems to be ca 2 months (8–10 weeks).
